# Combined Analysis of Expression Profiles in a Mouse Model and Patients Identified BHMT2 as a New Regulator of Lipid Metabolism in Metabolic-Associated Fatty Liver Disease

**DOI:** 10.3389/fcell.2021.741710

**Published:** 2021-11-11

**Authors:** Yongqiang Ma, Zhi Tan, Qiang Li, Wenling Fan, Guangshun Chen, Yangyang Bin, Yi Zhou, Junfang Yi, Xiaohua Luo, Jieqiong Tan, Zhongzhou Si, Jiequn Li

**Affiliations:** ^1^ Department of Liver Transplant, Second Xiangya Hospital, Central South University, Changsha, China; ^2^ Transplant Medical Research Center, Second Xiangya Hospital, Central South University, Changsha, China; ^3^ Department of Gastroenterology, The First Hospital of Changsha, Changsha, China; ^4^ Center for Medical Genetics, School of Life Science, Central South University, Changsha, China

**Keywords:** FXR, metabolic associated fatty liver disease, transcription analysis, BHMT2, PPAR γ

## Abstract

Metabolic associated fatty liver disease (MAFLD) is associated with obesity, type 2 diabetes mellitus, and other metabolic syndromes. Farnesoid X receptor (FXR, NR1H4) plays a prominent role in hepatic lipid metabolism. This study combined the expression of liver genes in FXR knockout (KO) mice and MAFLD patients to identify new pathogenic pathways for MAFLD based on genome-wide transcriptional profiling. In addition, the roles of new target genes in the MAFLD pathogenic pathway were also explored. Two groups of differentially expressed genes were obtained from FXR-KO mice and MAFLD patients by transcriptional analysis of liver tissue samples. The similarities and differences between the two groups of differentially expressed genes were analyzed to identify novel pathogenic pathways and target genes. After the integration analysis of differentially expressed genes, we identified 134 overlapping genes, many of which have been reported to play an important role in lipid metabolism. Our unique analysis method of comparing differential gene expression between FXR-KO mice and patients with MAFLD is useful to identify target genes and pathways that may be strongly implicated in the pathogenesis of MAFLD. The overlapping genes with high specificity were screened using the Gene Expression Omnibus (GEO) database. Through comparison and analysis with the GEO database, we determined that BHMT2 and PKLR could be highly correlated with MAFLD. Clinical data analysis and RNA interference testing *in vitro* confirmed that BHMT2 may a new regulator of lipid metabolism in MAFLD pathogenesis. These results may provide new ideas for understanding the pathogenesis of MAFLD and thus provide new targets for the treatment of MAFLD.

## Introduction

Metabolic-associated fatty liver disease (MAFLD), formerly known as non-alcoholic fatty liver disease (NAFLD), is the most common among chronic liver diseases. It is diagnosed based on histological evaluation (biopsy), imaging studies, or blood biomarker evidence of fat accumulation in the liver (hepatic steatosis), in addition to one of the following three criteria overweight/obesity, presence of type 2 diabetes mellitus (T2DM), or evidence of metabolic dysregulation (Eslam et al., 2020a). The pathogenesis and progression of MAFLD involve multiple factors, including insulin resistance, hormones secreted from adipose tissue, nutritional factors, gut microbiota, endoplasmic reticulum (ER) stress, innate immunity, genetic factors, and epigenetic factors (Tilg and Moschen, 2010; Buzzetti et al., 2016). MAFLD is a progressive disease. Most MAFLD patients have progressed from a simple steatosis to the more advanced form of the disease, which may ultimately lead to cirrhosis or hepatocellular carcinoma and liver decompensation. MAFLD is the most common cause of cirrhosis and the current leading indication for liver transplant in women and the second leading cause in men (Ogawa et al., 2019). In addition to liver transplantation, there are no effective therapies for advanced MAFLD that have been approved by the Federal Drug Administration (FDA) or European Medicines Agency (EMA); thus, it is crucial to explore the pathogenesis of MAFLD.

Farnesoid X receptor (FXR) is a ligand activated transcription factor that belongs to the nuclear receptor (NR) superfamily (Forman et al., 1995), and is mainly expressed primarily in the liver, intestine, and kidney, while bile acids (BAs) are its endogenous ligands. FXR mainly functions as the BA sensor by regulating genes that are critically involved in BA homeostasis, including BA biosynthesis, conjugation, and enterohepatic circulation (Sinal et al., 2000). Furthermore, FXR activation leads to the expression of various genes involved in glucose, lipid, lipoprotein metabolism, and bile acid synthesis (Kunne et al., 2014). Hepatic FXR can inhibit lipid uptake and synthesis and can enhance β-oxidation, thus reducing liver lipid accumulation. Therefore, FXR also plays a crucial role in the treatment of MAFLD. Obeticholic acid (OCA), a first-generation FXR agonist, has been applied in clinical practice and has achieved good clinical effects, the development of second-generation FXR agonists is currently ongoing (Ogawa et al., 2019). Nonbile acid FXR agonists with a high affinity for intestinal FXR have been developed as new-generation FXR agonists (Tully et al., 2017).

In the present study, we applied a unique analysis method to obtain 134 overlapping genes by comparing the differential gene expression in liver tissue of FXR-KO mice and MAFLD patients, and a series of signaling pathways and molecules have also been identified through differential gene expression analysis. Furthermore, in conjunction with the GEO datasets, we identified two specific overlapping genes BHMT2 and PKLR differentially expressed in both models. The correlation analysis of clinical data and RNA interference further confirmed the correlation between BHMT2 expression and hepatocyte lipid metabolism. The result of real-time PCR showed that BHMT2 could affect hepatocyte lipid metabolism by regulating PPARG expression. Thus, we propose BHMT2 as a potential regulator of lipid metabolism associated with MAFLD.

## Materials and Methods

### Clinical Samples and Patient Data

In this study, we included nine healthy control samples from adult patients who underwent surgical resection for hemangioma, and seven liver samples that were histologically diagnosed as MAFLD. Two experienced pathologists, blinded to clinical data, independently evaluated all liver samples according to the NAFLD activity score (NAS), defined as the sum of steatosis, inflammation, and ballooning of hepatocytes. Patients with a NAS score ≥5 were considered likely to have steatohepatitis (Kleiner et al., 2005). The clinical characteristics of the MAFLD and control groups are shown in Table 1.

**TABLE 1:** Clinical characteristics of MAFLD and control groups.

NO	Sex	Age (year)	BMI (kg/m^2^)	ALT (U/L)	ALP (U/L)	GGT (U/L)	TC (mmol/L)	TG (mmol/L)	HDL-C (mmol/L)	LDL-C (mmol/L)	FBG (mmol/L)	UA (μmol/L)	MetS	Diabetes	Hypertension	NAS
**MAFLD**																
1	M	42	37.2	183.7	110.5	119.4	6.67	2.45	1.14	3.97	5.5	543.64	+	−	+	6
2	M	40	30.5	49.4	93.7	78.4	6.62	2.07	0.95	4.18	6.2	518.76	+	+	+	5
3	M	37	30.4	25	56.7	44.7	5.68	1.98	0.92	2.75	6.8	481.7	+	+	−	5
4	M	46	22.5	100.7	98.4	113.4	4.98	1.54	1.26	2.78	6.7	456.78	−	+	+	4
5	M	50	22.2	25.3	47.6	48.6	6.02	1.93	1.04	3.65	7.3	506.71	+	+	+	5
6	M	54	27.8	27.8	47.4	38.8	5.1	1.68	1.21	2.87	5.1	437.14	−	−	+	4
7	M	45	32.7	56.4	67.8	73.4	5.45	1.86	0.95	3.41	5.8	387.43	+	+	−	5
**Control**																
A	M	37	25.4	30.7	92.7	42.7	4.86	1.44	1.26	2.76	4.3	478.7	−	−	−	2
B	M	53	23.9	25	44.7	67.5	4.43	1.64	1.41	2.78	5.8	407.5	−	−	−	1
C	M	48	23.7	24.6	61.8	55.4	4.93	1.57	1.27	2.64	5.5	421.7	−	−	−	1
D	M	31	21.5	49	51.7	78.6	5.74	1.87	1.56	2.86	4.9	367.3	−	−	+	3
E	M	54	23.9	8.7	51.3	27.8	4.51	1.34	1.54	2.47	6.3	398.7	−	+	−	2
F	M	48	23.1	11.9	48.7	37.4	4.61	1.56	1.31	2.54	4.9	367.1	−	−	−	0
G	M	52	16.3	35.7	47.8	65.8	4.57	1.68	1.45	2.55	4.5	417.2	−	−	+	1
H	M	49	24.2	33.7	76.3	34.1	4.94	1.68	1.47	2.73	4.9	373.2	−	−	−	1
I	M	29	22.1	38.5	37.4	44.5	4.6	1.37	1.36	2.89	4.6	376.8	−	−	−	0

Note: ALT, alanine aminotransferase; AST, aspartate aminotransferase; ALP, alkaline phosphatase; BMI, body mass index; FBG, fasting blood glucose; GGT, γ-glutamyl transpeptidase; HDL-C, high-density lipoprotein cholesterol; LDL-C, low-density lipoprotein cholesterol; MetS, metabolic syndrome; TC, total cholesterol; Tri, Triglycerides; UA, uric acid.

### Hepatic Transcriptome

Total RNA was extracted from liver tissue samples obtained from patients with MAFLD and healthy controls using the Illustra RNA spin Mini Kit (GE Healthcare, United States). RNA samples were quantified using a Nanodrop instrument (Thermo Fisher Scientific, United States) and qualified by agarose gel electrophoresis. Illumina kits which include procedures of RNA fragmentation, random hexamer primed first-strand cDNA synthesis, dUTP-based second strand cDNA synthesis, end-repairing, A-tailing, adaptor ligation, and library PCR amplification, were used for RNA-seq library preparation. Finally, the prepared RNA-seq libraries were qualified using a 2100 Bioanalyzer (Agilent, United States) and quantified using a qPCR absolute quantification method. The sequencing was performed using the Illumina Hi Seq 4000 platform. Raw sequencing data that passed the Illumina quality filter were used for the following analysis. Trimmed reads are aligned with the reference genome (Human GRCh38/hg38). Based on alignment statistical analysis (mapping ratio, rRNA/mt RNA content, and fragment sequence bias), we determined whether the results could be used for subsequent data analysis. After the livers of FXR-KO mice and control were obtained, hepatocyte RNA was extracted by the same method. Subsequently, expression profiling, differentially expressed genes, and differentially expressed transcripts were calculated. Genes with a *p-*value < 0.05 were considered differentially expression genes. Principal component analysis (PCA), correlation analysis, hierarchical clustering, gene ontology (GO), and pathway analysis were performed to explore the potential role of differentially expressed genes using R or the Python environment for statistical computing and graphical representation, respectively.

### Animal Studies

C57BL/6 mice were obtained from the Animal Research Center of Xiangya Medical College and we successfully constructed the FXR-knockout model mice (C57BL/6J FXR^-/-^ mice were generated using the CRISPR/Cas9 system). Eight-week-old male wild-type (WT) and FXR-KO mice were fed a high-fat diet (HFD) (Bio Serv) for 16 weeks. All mice were housed under specific pathogen-free and controlled temperature conditions with a 12-h light-dark cycle at 22–24°C. Only male mice were used for the experiments. All animal studies were approved by the Animal Care and Use Committee of Central South University and were carried out according to the Guide for the Care and Use of Laboratory Animals.

### Immunohistochemistry

Paraffin-embedded sections (5 µm) from each tissue were stained using immunohistochemistry with anti-BHMT2 or anti-PKLR antibodies (Sigma, United States). After deparaffinization, the sections were first incubated with 10% of normal goat serum in 50 mM Tris-HCl (pH 7.4) with 150 mM NaCl (TBS) at room temperature, followed by incubation with the primary antibody. The sections were then incubated with the biotinylated secondary antibody, the avidin-biotin-peroxidase complex (Vector Laboratories, United States), and 3,3′-diaminobenzidine in the presence of H_2_O_2_. All stained sections were photographed under a light microscope connected to a charge-coupled camera device.

### BODIPY and Immunofluorescence Staining

At 48 h post-transfection with siRNA, intracellular lipid droplets were stained with BODIPY 493/503 (Sigma, United States). The cells were washed with PBS and incubated in BODIPY 493/503 staining solution for 15 min at 37°C. The cells were then washed with PBS twice and fixed in 4% of paraformaldehyde for 15 min at room temperature, followed by permeabilization with 0.1% of Triton X-100. After blocking with 5% BSA for 30 min, the fixed cells were incubated with a Flag (M2) antibody (Sigma, United States) and detected using an Alexa Fluor-conjugated secondary antibody (Invitrogen, United States). The nuclei were counterstained with DAPI (Invitrogen, United States) for 2 min and washed with PBS three times again. Approximately, 10% of the cells with high or low fluorescence intensity were sorted and collected using a fluorescence-activated cell sorter (BD Bioscience, United States).

### Immunoblotting

CGI-58 antibody was purchased from Abcam. β-actin antibody was obtained from Cell Signaling Technology. Cells were lysed with lysis buffer containing 2% SDS, 62.5 mM Tris-HCl pH 6.8, and 10% glycerol. Protein concentrations were measured using a BCA kit and a protein standard. Subsequently, the samples were separated by SDS-PAGE and transferred to Immobilon-P polyvinylidene difluoride membranes (Millipore Corporation, Billerica, MA, United States). The membrane was blocked by incubating in 5% of defatted milk for 1 h, and incubated with the primary antibodies overnight at 4°C. After incubation, the membrane was washed three times for 30 min each and subsequently incubated with horseradish peroxidase-conjugated secondary antibodies. Visualization of target protein was achieved through Immobilon Western Chemiluminescent HRP substrate (Millipore Corporation, Billerica, MA, United States).

### Data and Statistical Analysis

Pathway enrichment and GO analysis were performed using the web application Metascape and DAVID with analyses including “GO Biological Processes” and “KEGG Pathway” as the default parameters. Protein-protein interaction (PPI) enrichment analysis was carried out using STRING and Cytoscape. The resultant network contained a subset of proteins that form physical interactions with at least one other member in the list. If the network contained between 3 and 500 proteins, the molecular complex detection (MCODE) algorithm was applied to identify the densely connected components of the network. Pathway and process enrichment analysis was applied to each MCODE component independently using Metascape, and the three best-scoring terms defined by the *p*-value were retained as the functional description of the corresponding components.

Then, we queried the GEO database from National Center for Biotechnology Information (NCBI) and extracted two data sets for analysis of gene expression in liver tissue of patients with MAFLD and in normal controls, we identified specific differentially expressed genes by comparing this list with the genes identified in this study, so as to further illustrate the importance of these overlapping genes in the pathogenesis of MAFLD.

Data are presented as means ± standard error (SEM). All data analyses were performed using Prism 8.3.0 (GraphPad Software Inc., San Diego, CA, United States). The raw data from each individual experiment were evaluated using an unpaired two-tailed *t-*test with 95% confidence interval (CI) in Prism. For data sets that did not pass the D’Agostino and Pearson omnibus normality test (*α* = 0.05), differences were evaluated using a two-tailed unpaired nonparametric Mann–Whitney test with 95% CI.

## Results

### FXR-KO Promoted the Development of MAFLD in Mice, but There Were no Significant Differences in FXR Expression Among MAFLD Patients

**FIGURE 1:**
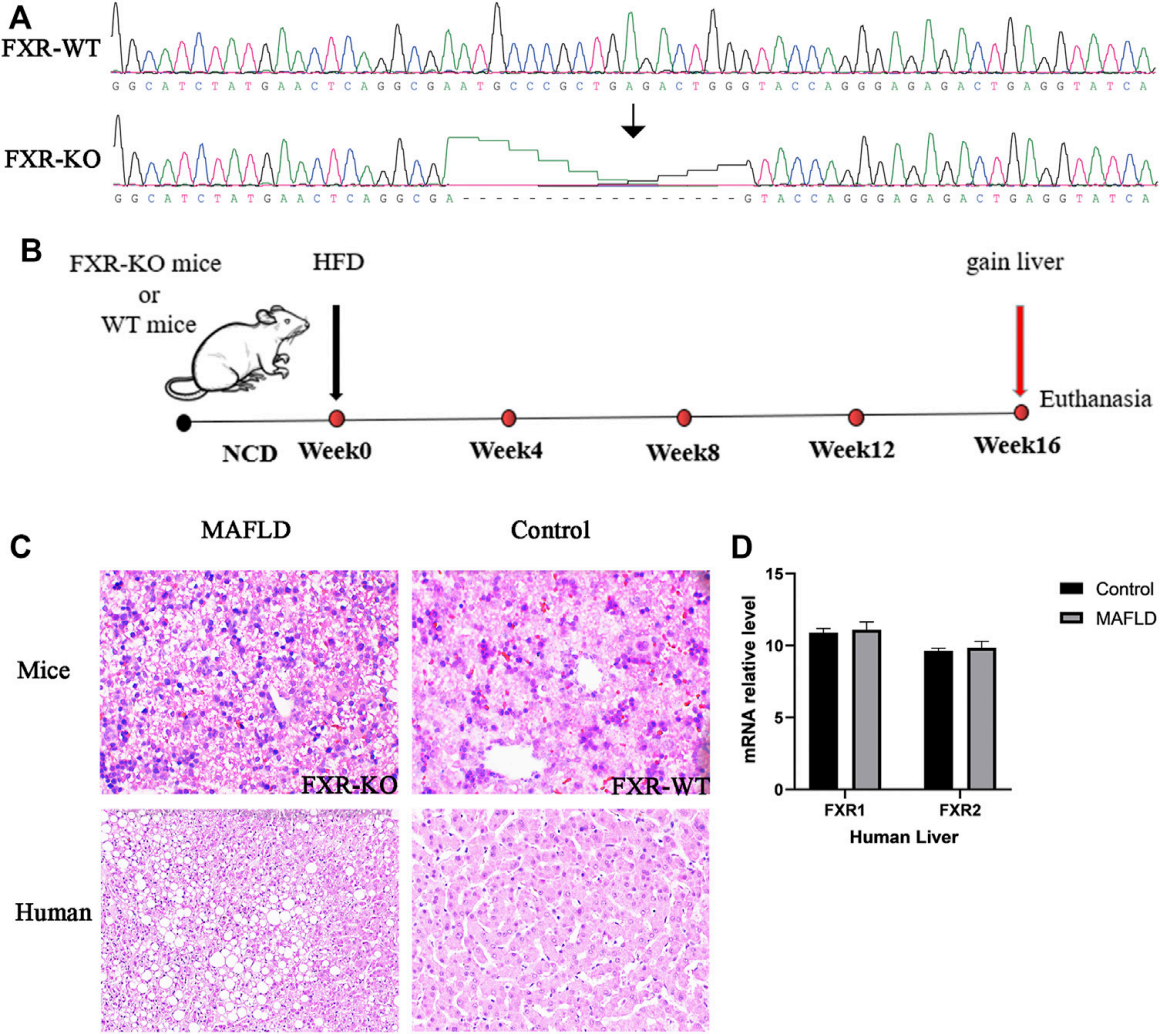
FXR is downregulated in the livers of MAFLD mice and is associated with increased lipid accumulation in the liver. **(A)** The result of gene sequencing in the liver of MAFLD mice and controls. **(B)** Schematic representation of the experimental design. **(C)** Paraffin-embedded liver sections stained with H&E. **(D)** Relative levels of FXR mRNA detected in the liver of MAFLD patients and controls.

To investigate the involvement of FXR in MAFLD, we used FXR^–/–^ and WT mice fed a HFD for 16 weeks to construct MAFLD models (Figures 1A,B). A shown in Figure 1C, the degree of steatosis and the ballooning of fat droplets were intensified in FXR^–/–^ MAFLD mice compared to WT MAFLD mice. Next, we analyzed the expression of FXR in liver tissues of nine normal controls and seven patients with a pathological diagnosis of MAFLD. There were no significant differences in FXR expression between the liver tissue of the MAFLD and control groups (Figure 1D *p* > 0.05). Consistent with a previous study, inhibition of FXR increased the degree of steatosis in animal models of MAFLD (Ma et al., 2013), which indicated inhibition of FXR was indeed involved in the pathogenesis of MAFLD. However, in the seven patients with MAFLD, we did not identify a significant relationship between FXR expression and MAFLD. Therefore, changes in FXR expression may not necessarily be associated with the onset of MAFLD.

**FIGURE 2:**
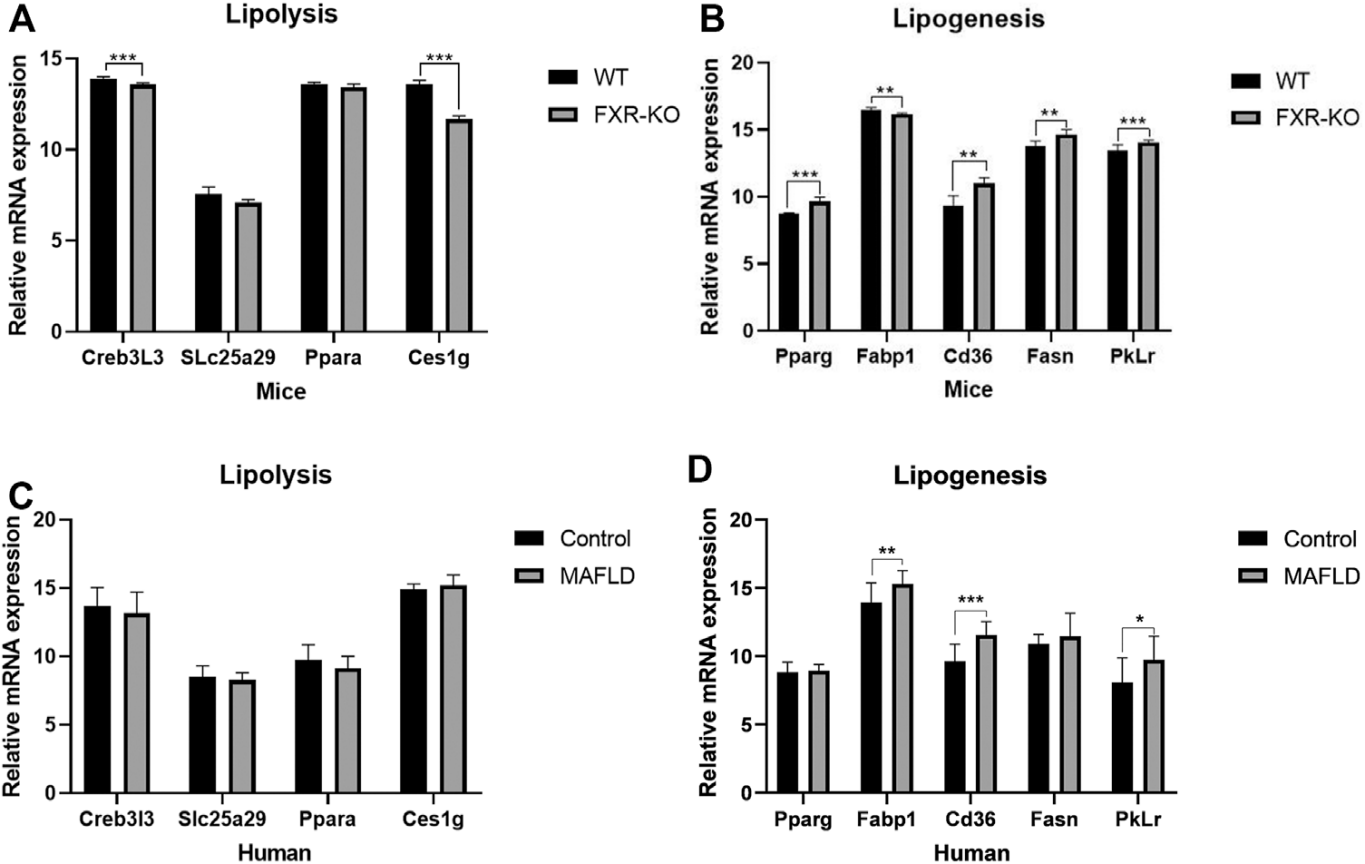
The expression of lipid metabolism genes was altered in patients/mice with MAFLD. **(A,B)** Expression of genes involved in *de novo* lipogenesis, β-oxidation, and lipolysis measured by qPCR. (*p < 0.05; **p < 0.01; ***p < 0.001).

To obtain further insight into steatohepatitis and to explore the pathogenesis of MAFLD at the gene level, we examined the transcriptional regulation of genes implicated in lipogenesis, β-oxidation, and lipolysis in the liver by previous studies (Correia et al., 2015). FXR-KO enhanced the expression of lipogenesis genes (PPARγ, Cd36, Fasn, Pklr, *p* < 0.05), decreased the expression of β-Oxidation genes (Creb3L3, SLc25a29, *p* < 0.05) and lipolysis gene (Ces1g, *p* < 0.05) (Figure 2A). We identified similar changes in liver gene expression in patients with MAFLD (Figure 2B). Therefore, changes in gene expression could represent driving factor of MAFLD. In mice, FXR deficiency resulted in an imbalance of liver lipid metabolism, which worsened lipid accumulation in the liver and further developed into steatohepatitis. In humans, FXR expression did not differ significantly across the 16 liver tissue samples. There may be a change in FXR protein activity, however, in addition to FXR, other genes or pathways which might play an important role in the pathogenesis of MAFLD have not yet been identified and need to be further explored.

### Pathway Analysis of Overlapping Genes and Differentially-Expressed Genes

**FIGURE 3:**
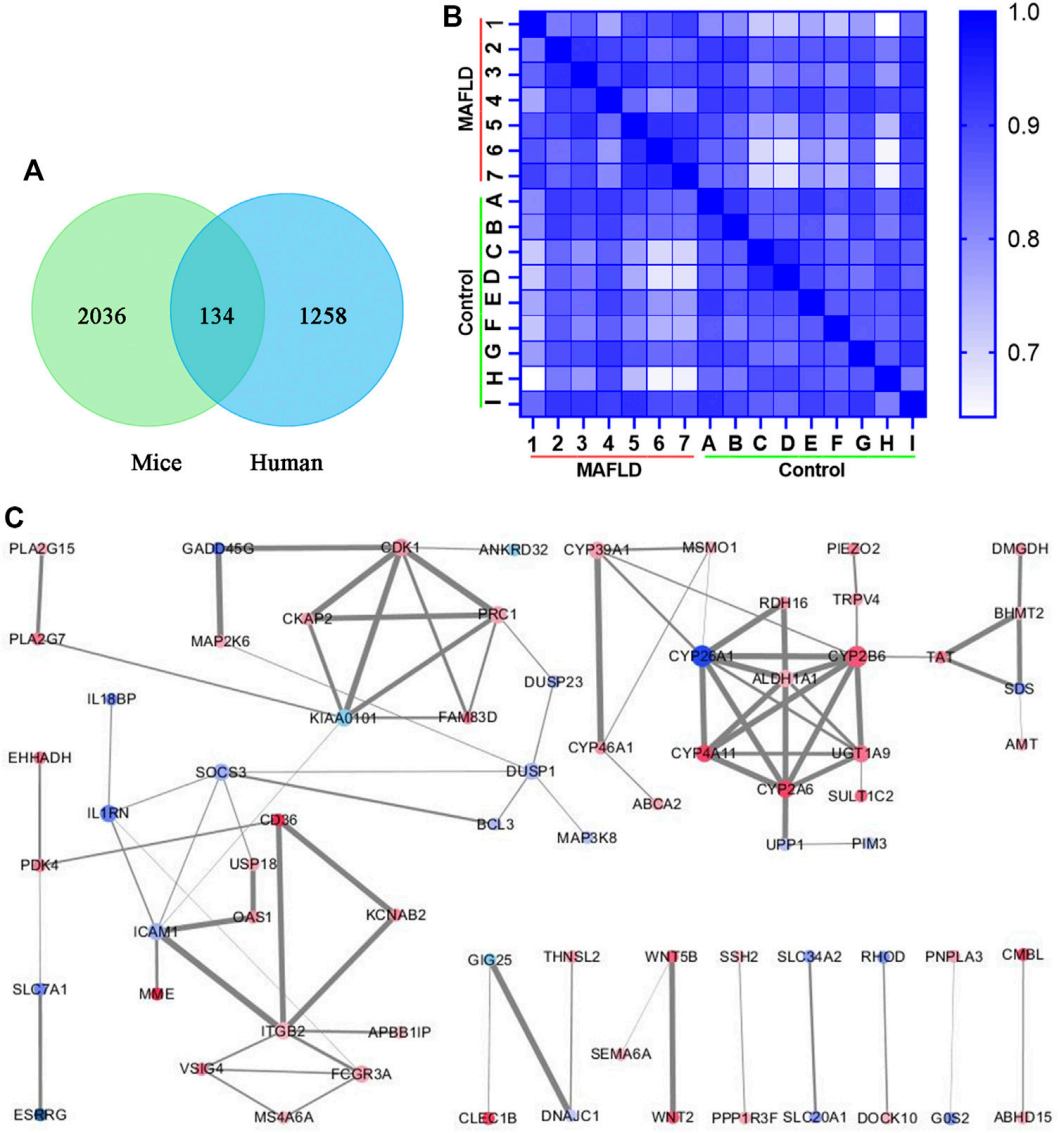
Overlapping genes that appeared in two groups of differentially expressed genes. **(A)** Venn diagram of genes that appear in both groups of differentially expressed genes. **(B)** Heatmap of overlapping genes from the transcriptomic analysis was compared with liver tissues of seven patients with MAFLD and nine normal controls. The lighter the color, the smaller the Pearson’s correlation coefficient. **(C)** PPI network from the Cytoscape network analysis of the overlapping genes. The size of the dot represents the number of related protein interactions, the thickness of the line represents the strength of the interaction between proteins. A darker red dot color represents the greater upregulation of gene expression, and a darker blue color represents a greater downregulation of gene expression.

To further explore the regulation of MAFLD, we compared the transcriptomic analysis of differentially expressed genes from liver tissues of three FXR-KO mice with MAFLD and three WT mice [*p* < 0.05, log_2_ (fold change) ≥0.5 or ≤−0.5] (Supplementary Table S1), the same transcriptomic analysis was applied to liver tissues of seven patients with MAFLD and nine normal controls [*p* < 0.05, log_2_ (fold change) ≥0.5 or ≤−0.5] (Supplementary Table S2). By comparing the two groups of differentially expressed genes, we identified 134 overlapping genes (Supplementary Table S3; Figure 3A). The heatmap shows Pearson’s correlation analysis of the expression data of the overlapping genes of liver tissues of MAFLD patients *versus* controls (Figure 3B). STRING and Cytoscape network analysis of the 134 overlapping genes is shown in Figure 3C.

**FIGURE 4:**
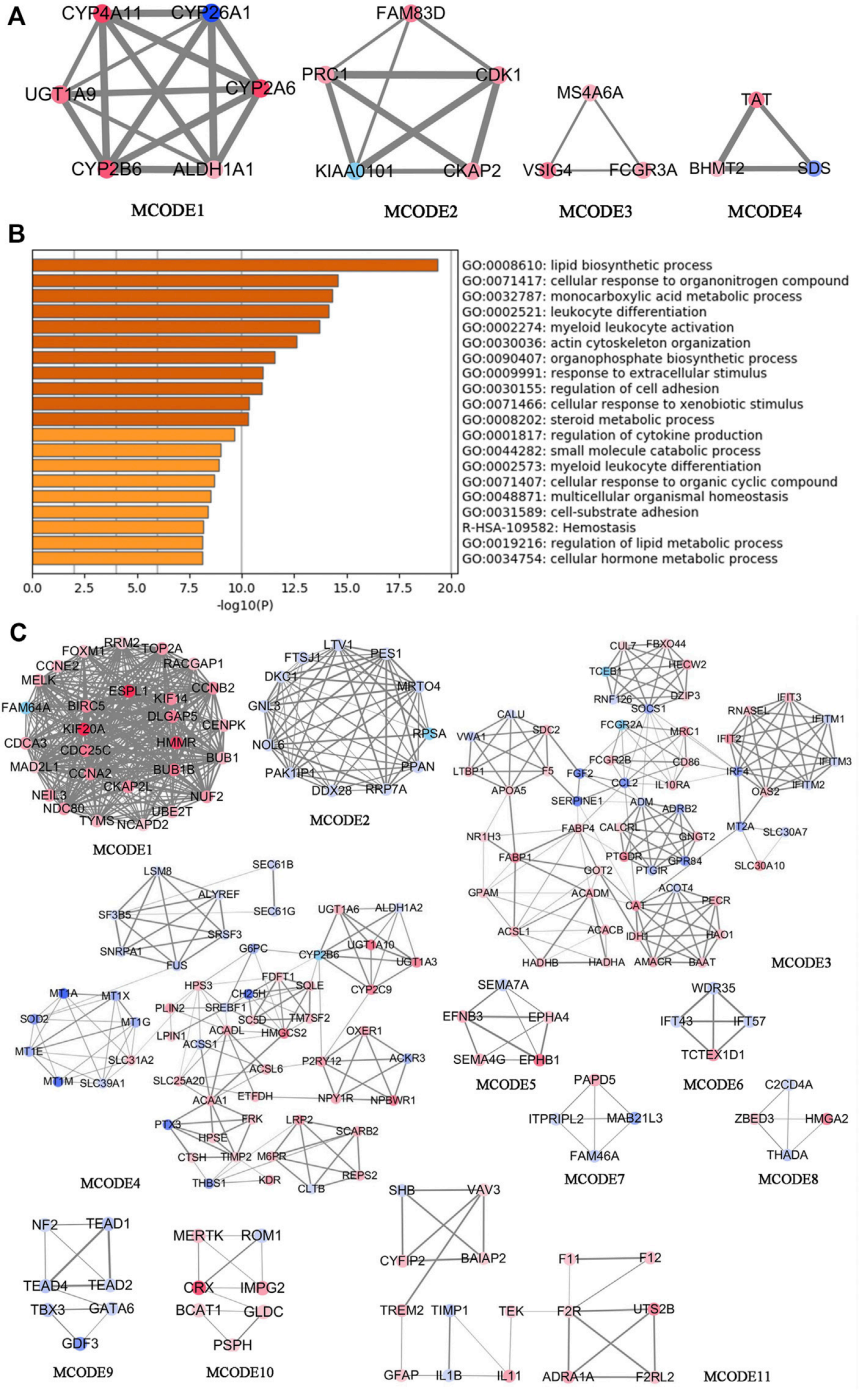
Comprehensive analysis of differentially expressed genes in the transcriptomic analysis of patients with MAFLD vs. control subjects using Metascape and Cytoscape. **(A)** The four significant MCODE components form the PPI network of overlapping genes. **(B)** Bar graphs showing the top 20 significantly enriched biological processes and pathways associated with MAFLD differently expressed genes excluding overlapping genes. **(C)** The top 11 significant MCODE components from the PPI network of MAFLD differently expressed genes excluding overlapping genes. The MCODE algorithm was applied to this network to identify neighborhoods where proteins are densely connected. Each node represents a protein, and the edge between nodes represents the interaction between two connected proteins.

DAVID was used for the GO and KEGG enrichment analysis of overlapping genes. The pathways and functions are listed in Table 2 (*p* < 0.05). The results suggested that these genes were enriched in metabolic pathways, retinol metabolism, oxidation-reduction process, lipid metabolic process, glucose homeostasis, and in insulin secretion. Next, we employed Cytoscape for PPI enrichment analysis to further understand protein interactions and used MCODE to identify densely connected network components. A list of important genetic components in the PPI network is shown in Figure 4A. Metascape was used for pathway and enrichment analyses for each MCODE component. The results showed that the biological functions of the MCODE components consisted of retinol metabolism, phase I-functionalization reactions of compounds, regulation of cell cycle processes, and cell division (Supplementary Table S4). Many of these overlapping genes have been reported to play an important role in lipid metabolism, which means these genes may be related to the onset of MAFLD. These findings suggested that our method of integrated analysis was reliable and feasible for identifying new regulating genes and pathways in MAFLD.

**TABLE 2:** DAVID functional annotation for overlapping genes.

Term	Count	*p*-value	Genes
**A. KEGG analysis**			
Metabolic pathways	22	0.001255141	DMGDH, SDS, PKLR, GDA, TAT, HDC, AMT, MSMO1, CMBL, PLA2G7, DPM2, CYP26A1, CYP2A6, CYP2B6, ETNK2, EHHADH, ALDH1A1, RDH16, MAN1C1, PNPLA3, UPP1, UGT1A9
Biosynthesis of antibiotics	8	0.002538832	SDS, PKLR, TAT, EHHADH, AMT, MSMO1, CMBL, PLA2G7
Retinol metabolism	6	2.37E-04	CYP26A1, CYP2A6, CYP2B6, ALDH1A1, RDH16, UGT1A9
TNF signaling pathway	5	0.014646592	SOCS3, BCL3, MAP3K8, ICAM1, MAP2K6
Glycine, serine, and threonine metabolism	3	0.046470689	DMGDH, SDS, AMT
**B. GO-BP analysis**			
**Term**	**Count**	***p*** **-** **value**	**Genes**
Apoptotic process	11	0.007898886	CSRNP1, SEMA6A, PEG10, ITGB2, CDK1, CKAP2, PIM1, PIM3, MAP3K8, GADD45G, MAP2K6
Oxidation-reduction process	11	0.010507529	CYP39A1, DMGDH, CYP26A1, CYP2A6, CYP2B6, ALDH1A1, CYP4A11, RDH16, MSMO1, KCNAB2, CYP46A1
Xenobiotic metabolic process	6	2.48E-04	SULT1B1, CYP26A1, CYP2B6, CMBL, UGT1A9, CYP46A1
Transmembrane transport	6	0.031793828	SLC5A6, SLC14A1, ABCA2, SLC16A12, SLC25A25, TOMM40L
Lipid metabolic process	5	0.026915673	PLA2G15, ABCA2, IL1RN, RDH16, CD36
Metabolic process	5	0.033337445	EHHADH, MAN1C1, UGT1A9, HDHD3, SULF2
Steroid metabolic process	4	0.003650733	SULT1B1, CYP2A6, CYP2B6, MSMO1
Response to mechanical stimulus	4	0.00884836	P2RX7, TRPV4, IGFBP2, PIEZO2
Response to glucocorticoid	4	0.011524662	IL1RN, DUSP1, TAT, IGFBP2
Glucose homeostasis	4	0.03653111	PPP1R3G, OAS1, TRPV4, PDK4
Insulin secretion	3	0.020895581	IL1RN, ILDR2, RAPGEF4
Epoxygenase P450 pathway	3	0.007306425	CYP2A6, CYP2B6, CYP4A11
Regulation of G-protein coupled receptor protein signaling pathway	3	0.032082211	RIC8B, RGS16, RAMP1
Cellular amino acid metabolic process	3	0.033614492	SDS, TAT, HDC
Extrinsic apoptotic signaling pathway	3	0.036762954	P2RX7, TNFRSF12A, G0S2
Receptor internalization	3	0.038378184	ITGB2, CD36, RAMP1
T cell activation via T cell receptor contact with antigen bound to MHC molecule on antigen presenting cell	2	0.021463309	APBB1IP, ICAM1
Positive regulation of cytoskeleton organization	2	0.028515721	P2RX7, SORBS3
Pyruvate biosynthetic process	2	0.04246967	SDS, PKLR
Positive regulation of prostaglandin secretion	2	0.04937192	P2RX7, MAP2K6

To discover new target genes and pathways unrelated to FXR, excluding overlapping genes, we obtained 1,124 differentially expressed genes from transcriptomic analysis of liver tissues of MAFLD patients vs. controls [*p* < 0.05, log_2_ (fold change) ≥0.5 or ≤−0.5] (Supplementary Table S5). We used Metascape to conduct GO and KEGG enrichment analyses (Supplementary Table S6; Figure 4B) (Zhou et al., 2019). The top 20 significant pathways and functions are listed in accordance with their *p*-values in Figure 4B. The results suggested that these genes are involved in monocarboxylic acid metabolic processes, regulation of lipid metabolic processes, carbohydrate metabolic processes, the PPAR signaling pathway, fatty acid transmembrane transport, and triglyceride metabolic processes. We also employed Cytoscape for PPI enrichment analysis to investigate protein interactions and used the MCODE algorithm to identify densely connected network components. A list of important genetic components in the PPI network is shown in Figure 4C. The 11 most important MCODE components were selected, after which pathway and enrichment analyses were applied by Metascape to each MCODE component (Supplementary Table S7). The results showed that the biological functions of the MCODE components included protein binding, cell division, rRNA processing, the PPAR signaling pathway, fatty acid metabolism, fatty acid degradation, transmembrane-ephrin receptor activity, metabolic pathways, and DNA binding. These pathways and biological functions may play an important role in the pathogenesis of MAFLD. The identification of a new regulator of lipid metabolism associated with MAFLD, would be of great significance in improving the understanding of the pathogenesis of MAFLD.

### Expression of BHMT2 and PKLR was Elevated in Patients With MAFLD

**FIGURE 5:**
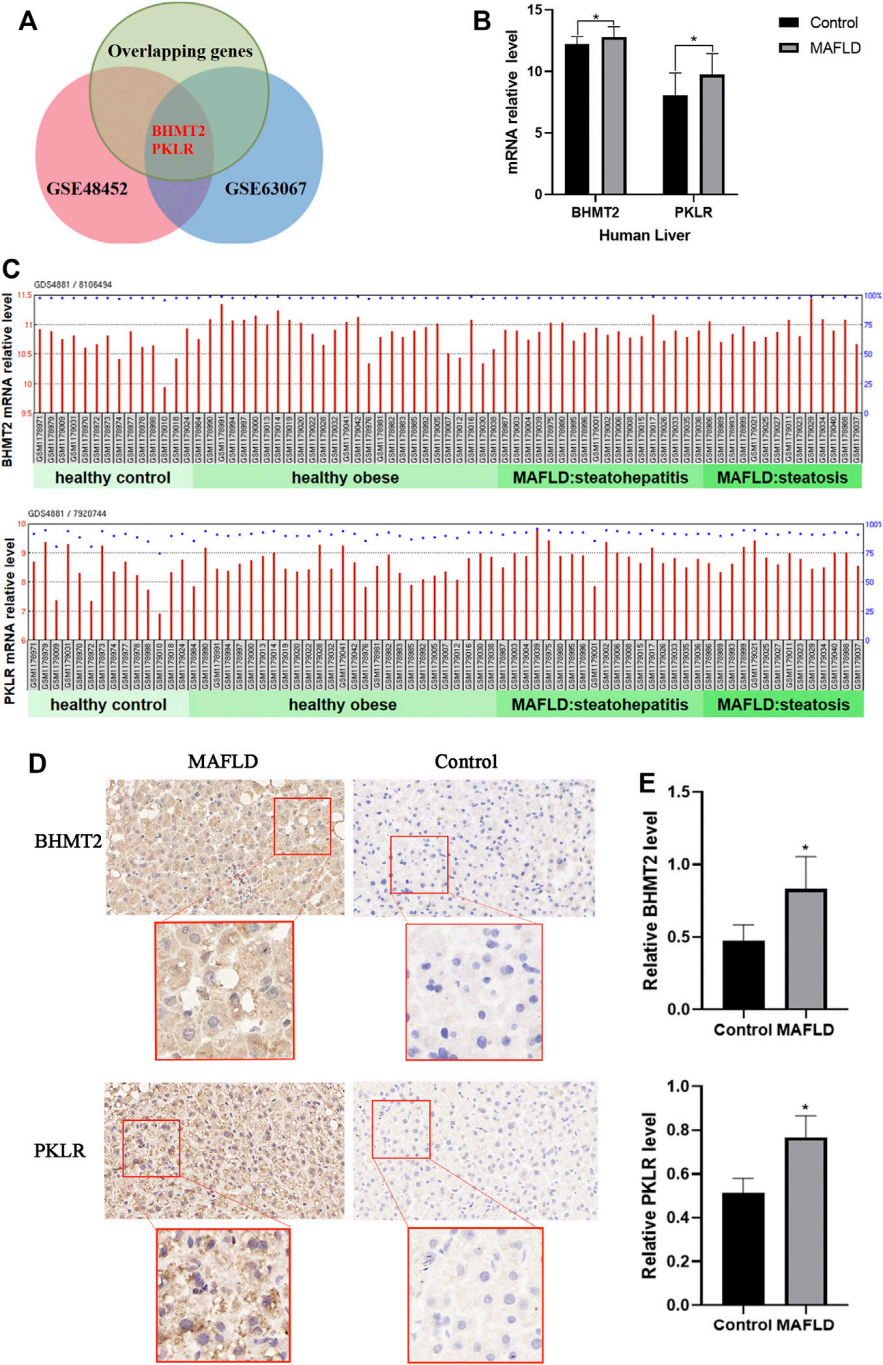
Expression of BHMT2 and PKLR was elevated in patients with MAFLD **(A)** Venn diagram of genes appearing in the three groups of differentially expresses genes. **(B)** mRNA relative levels of BHMT2 and PKLR detected in the liver of MAFLD patients and controls in the GSE48452 dataset. **(C)** mRNA relative levels of BHMT2 and PKLR detected in the liver of MAFLD patients and controls. **(D)** Immunohistochemistry assay of BHMT2 and PKLR in liver tissues of patients with MAFLD and controls. **(E)** Statistical results of the relative expression of BHMT2 and. PKLR. The relative level of BHMT2 and. PKLR were determined by calculating the average intensity of each sample normalized to the average intensity of the control sample. (*p < 0.05; **p < 0.01; ***p < 0.001).

Next, we verified the reliability of the above differentially expresses gene with datasets from the GEO database, choosing the GSE48452 dataset from the study of “Human liver biopsy of different phases from control to NASH” (Ahrens et al., 2013). A total of 14 healthy controls, 14 steatosis (SS) samples, and 18 nonalcoholic steatohepatitis (NASH) samples were included in the GSE48452 dataset (Liu et al., 2020). According to the transcriptomic analysis, we identified the differentially expressed genes in liver tissues of the GSE48452 dataset (Supplementary Table S8). In the GSE63067 dataset, differentially expressed genes were stratified according to different stages of NAFLD, and included genome-wide expression patterns from two cases of human steatosis and nine cases of human NASH and seven healthy controls (Supplementary Table S9). These two lists of differentially expressed genes were compared with the 134 overlapping genes, and two overlapping genes were identified: BHMT2 and PKLR (Figures 5A,B). Our results of the transcriptional analysis comparing liver tissues of MAFLD patients and controls indicated the expression of BHMT2 and PKLR was elevated in MAFLD patients (*p* < 0.05) (Figure 5C).

To further confirm the changes in the expression of BHMT2 and PKLR in the liver of patients with MAFLD, we also performed immunohistochemical staining using these 16 liver tissue samples, The results showed that the expression of BHMT2 and PKLR was significantly increased in liver tissues of MAFLD patients compared to controls (Figures 5D,E).

**FIGURE 6:**
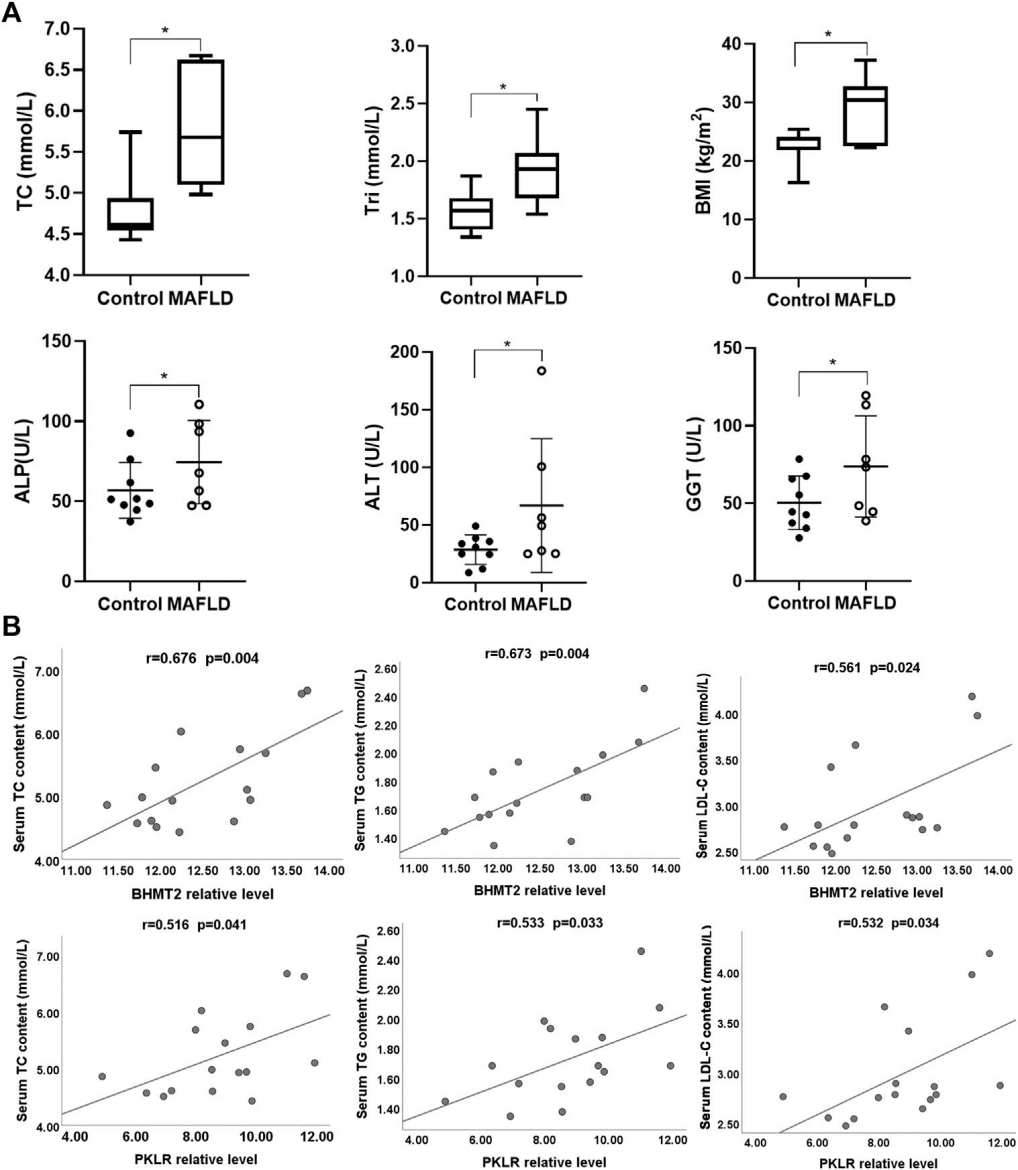
Analysis of BHMT2 and PKLR with clinical parameters. **(A)** Comparative analysis of clinical indicators between MAFLD patients and controls. **(B)** Correlation analysis between BHMT2, PKLR with serum TC, TG, and LDL-C levels. BMI, body mass index; ALT, alanine aminotransferase; ALP, alkaline phosphatase; GGT, γ-glutamyl transpeptidase; TC, total cholesterol; Tri (TG), Triglycerides. (*p < 0.05; **p < 0.01; ***p < 0.001).

Then, we explored the functions of BHMT2 and PKLR in MAFLD. We analyzed the clinical characteristics of seven patients with NASH and nine normal controls. The results showed that the clinical parameters BMI, TC, TG, ALT, ALP, and GGT of patients with MAFLD were higher than those of controls (Figure 6A). According to the latest clinical practice guidelines of the Asian Pacific Association for the Study of the Liver (APASL) on MAFLD (Eslam et al., 2020b), these indicators play an important role in the diagnosis of MAFLD. We questioned whether there was any relationship between the change in these clinical indicators with the expression level of BHMT2 and PKLR. Pearson’s correlation analysis was performed and the results indicated that the expression levels of BHMT2 and PKLR were positively correlated with the serum content of TC, TG, and LDL-C (Figure 6B). Overall, these findings suggested that BHMT2 and PKLR could be involved in the pathogenesis of MAFLD.

### BHMT2 May be Involved in Hepatocyte Lipid Metabolism by Regulating the Expression of PPARG *In Vitro*.

**FIGURE 7:**
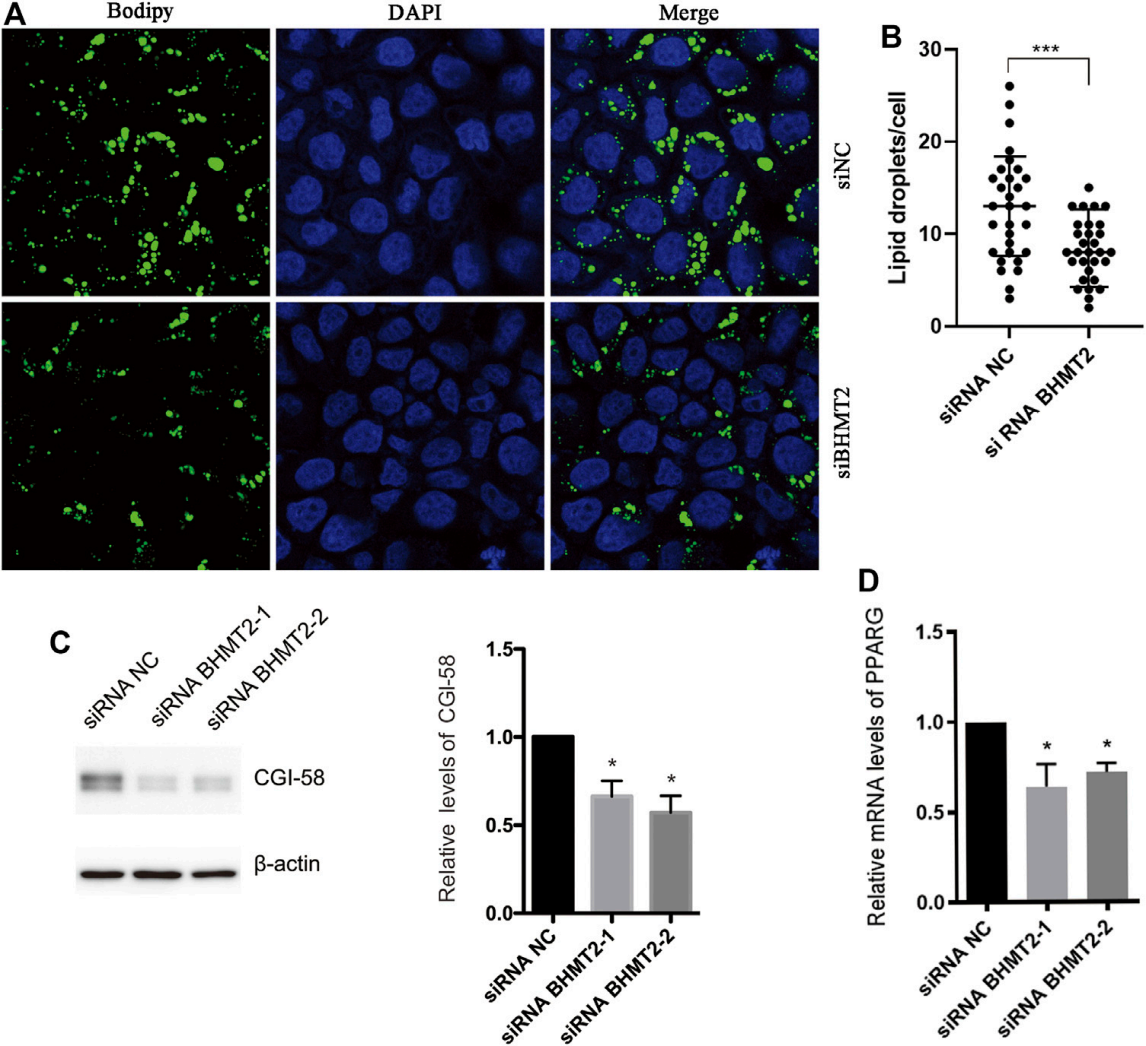
BHMT2 may be involved in hepatocyte lipid metabolism by regulating the expression of PPAR *in vitro*. **(A)** L02 cells were transfected with BHMT2 siRNA and control siRNA. **(B)** The number of lipid droplets per cell shown in **(A)** was quantified. **(C)** Western blotting results of CGI-58, β-actin is as a loading control. **(D)** PCR results of PPARG. (*p < 0.05; **p < 0.01; ***p < 0.001).

Previous studies have indicated that PKLR plays an important role in the pathogenesis of MAFLD (Yki-Järvinen, 2014; Byrne and Targher, 2015). We questioned what role BHMT2 could play in the development of MAFLD. To further verify the function of BHMT2 in lipid metabolism in the liver, we modified the expression of BHMT2 in L02 cells by RNAi. When BHMT2 was downregulated, the presence of OA (oleic acid) + PA (palmitic acid)-induced lipid droplets (LD) was significantly decreased (Figures 7A,B). We used CGI-58 as lipid drop markers for the western blotting semi-quantitatively analysis, and the results showed that inhibition of BHMT2 expression reduced the expression of CGI-58. It further confirmed the effect of BHMT2 on lipid metabolism *in vitro* (Figure 7C). The results of real-time PCR revealed that inhibition of BHMT2 expression resulted in decreased PPARG expression (Figure 7D), which confirmed that BHMT2 may be involved in the metabolism of hepatocyte lipids by regulating the expression of PPARG *in vitro.*

## Discussion

In this study, using a unique analytical approach, we comprehensively analyzed the regulatory factors of MAFLD at the genome-wide level. We compared the differentially expressed genes in FXR-KO mice and patients with MAFLD and their respective controls, and identified 134 overlapping genes. These genes were found to be involved in important signaling pathways and in lipid metabolism, as identified by pathway analysis, which also demonstrated the reliability of our unique analytical method. Using a similar approach, in conjunction with the GEO database, we identified two specific overlapping genes BHMT2 and PKLR from an independent human dataset of MAFLD. Herein, we demonstrated that BHMT2 is a newly identified regulator of lipid metabolism associated with MAFLD.

In previous studies, genome-wide association studies identified dozens of genes with multiple polymorphisms that were associated with the increased risk of developing fatty liver disease in specific populations, including PPARG, PNPLA3, TM6SF2, PCSK9, HSD17B13, FXR, GCKR, APOB, LPIN1, UCP2, IFLN4, and PKLR (Yu et al., 2016; Lee et al., 2017; Schumacher and Guo, 2019). Related studies have shown that FXR plays a precise role in the occurrence and development of MAFLD. FXR agonism has been shown to be a promising pharmacological target, FXR activation is protective against liver inflammation associated with NASH (Armstrong and Guo, 2017). The expression of factors in both the adaptive and innate immune response in the liver is regulated in an FXR-dependent and independent manner. In human hepatocytes, FXR upregulated peroxisome proliferator-activated receptor alpha (PPARα) levels, which subsequently increased fatty acid oxidation (Verbeke et al., 2016). Furthermore, mouse livers deficient in FXR, exhibited elevated serum cholesterol levels compared to WT mice when fed a high-cholesterol diet (Pineda Torra et al., 2003).

We used FXR-KO mice to construct MAFLD models. The results of H&E staining indicated that decreasing the expression of FXR in the liver could lead to the development of MAFLD. In humans, FXR expression was not significantly different between MAFLD and controls, which could reflect a change in FXR protein activity, and conversely may also indicate that changes in FXR expression may not be necessarily associated with the onset of MAFLD. Besides FXR, other genes or pathways have yet to be identified.

We performed a transcriptomic analysis using liver tissues from FXR-KO mice and MAFLD patients, and identified 134 overlapping differentially expressed genes in the two models. Pathway analysis suggested that these genes were enriched in metabolic pathways, retinol metabolism, oxidation-reduction processes, lipid metabolic processes, glucose homeostasis, and in insulin secretion. The PPI enrichment analysis showed that the biological functions of the MCODE components consisted of retinol metabolism, phase I functionalization modification of compounds, regulation of cell cycle processes, and cell division. FXR is associated with several signaling pathways besides lipid metabolism, which indicates that changes in these signal pathways and genes are downstream events or results that lead to MAFLD. Therefore, the overlapping genes may play an important role in the pathogenesis of MAFLD, which needs to be further studied.

To identify new target genes and pathways that are independent of the FXR pathway, we analyzed the pathway enrichment involving the identified 1,124 differentially expressed genes. Our results suggested that these genes were involved in monocarboxylic acid metabolic processes, regulation of lipid metabolic processes, carbohydrate metabolic processes, the PPAR signaling pathway, fatty acid transmembrane transport, and triglyceride metabolic process. The PPI enrichment analysis showed that the biological functions of the MCODE components involved protein binding, cell division, rRNA processing, the PPAR signaling pathway, fatty acid metabolism, fatty acid degradation, transmembrane-ephrin receptor activity, metabolic pathways, and DNA binding. These pathways and biological functions may also play an important role in the pathogenesis of MAFLD.

We next combined the GEO databases GSE48452 and GSE63067 from NCBI, and identified BHMT2 and PKLR among the 134 overlapping genes. Our own transcriptional analysis showed that the expressions of BHMT2 and PKLR was elevated in MAFLD. Immunofluorescence staining further showed that the expression of BHMT2 and PKLR was significantly increased in MAFLD compared to controls. Previous studies showed that the protein encoded by pyruvate kinase L/R (PKLR) is a kinase that catalyzed the transphosphorylation of phosphoenolpyruvate into pyruvate and ATP, which are the rate-limiting steps of glycolysis. Defects in this enzyme, due to gene mutations or genetic variations, are common causes of chronic hereditary nonspherocytic hemolytic anemia. Most importantly, the modulation of these genes affects key metabolic pathways associated with lipid metabolism (i.e., steroid biosynthesis, the PPAR signaling pathway, fatty acid synthesis, and oxidation) and have been proposed to be involved in the progression of MAFLD. For example, inhibition of PKLR led to decreased glucose uptake and decreased mitochondrial activity in HepG2 cells (Schmitt et al., 2015). *In vivo* knockdown experiments of PKLR improved both steatosis and insulin resistance (Liu et al., 2019). Furthermore, PKLR induced mitochondrial stress in both steatosis and fibrosis models, and silencing PKLR relieved this stress and promoted the resolution of NAFLD/NASH (Chella Krishnan et al., 2018). Therefore, PKLR could be considered an efficient treatment strategy for MAFLD.

The protein encoded by BHMT2 is a methyl transferase that can catalyze the transfer of the methyl group from betaine to homocysteine. Anomalies in homocysteine metabolism have been implicated in disorders ranging from vascular disease to neural tube birth defects such as spina bifida (Chella Krishnan et al., 2021). BHMT2 is involved in adolescent obesity by affecting the metabolism of amino acids, that may be candidate genes in the etiology of obesity (Mostowska et al., 2010). BHMT2 has been reported to be downregulated during both short- and long-term weight loss (Aguilera et al., 2015; Bollepalli et al., 2018). BHMT overexpression increases PtdCho synthesis, leading to reduced lipid accumulation in the liver, while BHMT deficiency leads to fatty liver (Ji et al., 2008). In addition, BHMT^-^/^-^ mice presented reduced adiposity, enhanced insulin sensitivity, glucose tolerance, and increased whole body energy expenditure (Teng et al., 2011; Teng et al., 2012). BHMT may be related to lipid metabolism, but there have been no reports indicating that BHMT2 is involved in the lipid metabolism observed in MAFLD pathogenesis.

In the present study, the evaluation of the clinical characteristics showed that the BMI, TC, Tri, ALT, ALP, and GGT levels of patients with MAFLD were higher than those of controls. Pearson’s correlation analysis indicated that BHMT2 and PKLR were positively correlated with serum levels of TG, TC, and LDL-C. Similarly, our RNAi studies and western blotting showed that reducing BHMT2 expression could significantly reduce hepatocyte LD accumulation *in vitro*. Real-time PCR results showed that inhibition of BHMT2 expression resulted in decreased PPARG expression. The PPAR signaling pathway as an important pathway of lipid metabolism plays an crucial role in the pathogenesis of MAFLD (Pawlak et al., 2014; Montagner et al., 2016). Peroxisome proliferator-activated receptor (PPAR)α, β/δ, and γ modulate lipid homeostasis. Whole-body and hepatocyte-specific PPARα-deficient mice develop aggravated liver steatohepatitis when fed a HFD and or a methionine and choline-deficient die (Wang et al., 2020). Activation of PPARβ/δ may prevent dyslipidemia, insulin resistance, obesity, and NAFLD (Palomer et al., 2018). PPARG is highly expressed in adipose tissue and macrophages, and plays important roles in adipogenesis, lipid metabolism, insulin sensitivity, and immune regulation (Ahmadian et al., 2013). When PPARγ is ectopically overexpressed in hepatocytes, lipid droplets emerge. Adenovirus-mediated overexpression of PPARγ2 in hepatocytes increased hepatosteatosis and hepatocyte-specific disruption of PPARγ gene (PPARG) decreased liver steatosis in PPARγ^−^/^−^ mice (Yu et al., 2003). Therefore, BHMT2 may be involved in hepatocyte lipid metabolism by regulating the expression of PPARG *in vitro*. However, the detailed mechanisms involved in the reduction of LD reducing induced by BHMT2 deficiency requires further study.

### RNA Interference

The RNA oligos complementary to the BHMT2 gene and nontarget sites were obtained from GenePharma (Shanghai, China). OligoRNA was introduced into L02 cells by transfection with Lipofectamine 2000 reagent (Invitrogen, United States) according to the protocol provided by the manufacturer. The human liver cell line L02 (Pang et al., 2000) was obtained from the Type Culture Collection of the Chinese Academy of Sciences (Shanghai, China) and was cultured in RPMI-1640 medium containing 20% FBS (Invitrogen, United States) with 4 mM of L-glutamine, and 10 μg/ml of penicillin and streptomycin at 37°C, in an atmosphere of 5% CO_2_.

## Conclusion

In summary, our study comprehensively analyzed the regulatory factors of MAFLD at the genome-wide level by comparing the differentially expressed genes of FXR-KO mice and patients with MAFLD. Through this unique analysis method, we identified many new target genes and pathways that may potentially play an important role in the pathogenesis of MAFLD. Most importantly, we demonstrated that BHMT2 is a new regulator of lipid metabolism and is involved in MAFLD pathogenesis. Overall, our results may provide a better understanding of the pathogenesis of MAFLD and thus provide new targets for the treatment of MAFLD.

## Data Availability

The datasets presented in this study can be found in online repositories. The names of the repository/repositories and accession number(s) can be found below: GEO, GSE183229.
